# Prevalence of Fascioliasis in Livestock and Humans in Pakistan: A Systematic Review and Meta-Analysis

**DOI:** 10.3390/tropicalmed7070126

**Published:** 2022-07-07

**Authors:** Muhammad Rizwan, Mobushir Riaz Khan, Muhammad Sohail Afzal, Hajra Manahil, Sobia Yasmeen, Muhammad Jabbar, Shumaila Irum, Sami Simsek, Samia Wasif, Tahir Mahmood, Haroon Ahmed, Jianping Cao

**Affiliations:** 1 Department of Biosciences, COMSATS University Islamabad (CUI), Islamabad 45550, Pakistan; fa19-pbm-002@student.comsats.edu.pk (M.R.); manahilishaq08@gmail.com (H.M.); sobiayasmeen489@gmail.com (S.Y.); 2 School of Agricultural, Environmental and Veterinary Sciences, Charles Sturt University, Albury, NSW 2640, Australia; mobkhan@csu.edu.au; 3 Department of Life Sciences, University of Management & Technology (UMT), Lahore 54770, Pakistan; sohail.afzal@umt.edu.pk; 4 Department of Statistics, University of Gujrat, Gujrat 50700, Pakistan; muhammad.jabbar@uog.edu.pk; 5 Department of Mathematics and Statistics, Université Laval, Quebec City, QC G1V 0A6, Canada; 6 Department of Zoology, University of Gujrat, Gujrat 50700, Pakistan; shamaila.irum@uog.edu.pk; 7 Department of Parasitology, Faculty of Veterinary Sciences, Firat University, Elazig 23119, Turkey; ssimsek@firat.edu.tr; 8 Department of Humanities, COMSATS University Islamabad (CUI), Islamabad 45550, Pakistan; samia.wasif@comsats.edu.pk; 9 Industrial and Systems Engineering Department, College of Computing and Mathematics, King Fahd University of Petroleum and Minerals, Dhahran 31261, Saudi Arabia; tahir.mahmood@kfupm.edu.sa; 10 Interdisciplinary Research Center for Smart Mobility & Logistics, King Fahd University of Petroleum and Minerals, Dhahran 31261, Saudi Arabia; 11 National Institute of Parasitic Diseases, Chinese Center for Disease Control and Prevention (Chinese Center for Tropical Diseases Research), Shanghai 200025, China; 12 Key Laboratory of Parasite and Vector Biology, National Health Commission of the People’s Republic of China, World Health Organization Collaborating Center for Tropical Diseases, Shanghai 200025, China; 13 The School of Global Health, Chinese Center for Tropical Diseases Research, Shanghai Jiao Tong University School of Medicine, Shanghai 200025, China

**Keywords:** fascioliasis, prevalence, livestock, human, meta-analysis, Pakistan

## Abstract

Fascioliasis is a parasitic infection that affects both livestock and humans. Understanding the distribution of *Fasciola* spp. can help the development of preventive measures to control fascioliasis. This systematic review and meta-analysis aimed to estimate the status of fascioliasis among livestock and humans in Pakistan between 2000 and 2020. Based on the selection criteria, 25 articles were selected from Google Scholar, PubMed, and Scopus. This review included 76,099 animals, including 13,738 that were positive for fascioliasis. The overall prevalence was 18.1%; it was 0.3% in humans and 20.1% in livestock. Among animal hosts, the prevalence was highest in sheep (53.5%), followed by the goats (44.9%), cows (21.3%), buffaloes (16.8%), cattle (12.7%), and humans (0.3%). Sindh had the highest prevalence at 42.7%, followed by Baluchistan (25.2%), Punjab (17.7%), Khyber Pakhtunkhwa (10.7%), and Islamabad capital territory (1.5%). In the Punjab province, sheep had the highest prevalence (65.7%); in Khyber Pakhtunkhwa, it was buffalo (15.9%); and in Baluchistan, it was cows (28.5%). The prevalence of *Fasciola* spp. was higher in Sindh and Baluchistan than in the other provinces. The presented results are essential for developing preventive approaches for the management of human health and minimizing economic loss in the livestock industry in Pakistan. Preventive-curative treatments two times a year followed by a prophylactic treatment at the end of the dry season are crucial throughout the areas of Pakistan that serve as hotspots for infection by Fasciola sp. For humans, regular, prioritized surveys must be performed for high-risk populations so that the real situation can be assessed and addressed in a timely manner.

## 1. Introduction

Fascioliasis is a parasitic disease caused by *Fasciola* spp. and is well recognized because of its immense impact on the health of host animals. Fascioliasis is transmitted to herbivorous animals and humans through the consumption of contaminated water and green vegetables [1]. According to the World Health Organization, fascioliasis affects at least 17 million individuals in more than 70 countries around the globe [2]. Further, *Fasciola* spp. causes huge economic losses in the livestock industry by affecting meat and milk production [3]. Fascioliasis in livestock is characterized by liver seizures, low reproductive capability, and delays in diagnosis that are associated with high death rates [3]. The main causative agents of fascioliasis are *Fasciola gigantica* and *Fasciola hepatica* [4]. *F. gigantica* is common in Asia and Africa, while *F. hepatica* is spread across all the continents. Both species follow two host life cycles: (1) that of the Lymnaeidae family of fresh-water snails that act as vectors or intermediates, and (2) that of large-size herbivorous species and a wide range of mammals including humans [5]. Human fascioliasis may be foodborne, waterborne, or both. Foodborne human fascioliasis is caused by the ingestion of wild plants and vegetables, while waterborne human fascioliasis is caused by the consumption of parasite-contaminated water [6]. However, to date, there has been very little research on the transmission of fascioliasis in human beings [4].

The most common techniques for the diagnosis of fascioliasis include the direct parasitological method (identification of eggs in the feces of animals and in duodenal or bile samples for human fascioliasis. Microscopic examination of eggs has high specificity and sensitivity, while direct smear analysis is useful for the rapid detection and classification of helminth eggs [7,8]. However, accurate and species-specific identification of eggs remains a challenge because about 59 types of intestinal fluke cause various parasitic foodborne diseases in Southeast Asian regions. As the eggs of these parasitic species are morphologically similar, it is difficult to accurately identify the species-specific eggs from feces [5,9,10]. Instead, the PCR method can be used for differentiation of *Fasciola* species [11,12], and specific *Fasciola*-specific antigens can also be identified with ELISA or immunoblotting [13].

The prevalence of fascioliasis has been reported in various continents. Ten studies from Mexico [14,15], Argentina [16,17], Brazil [18], Peru [19], and Colombia [20] showed that the prevalence of fascioliasis in these five countries between 2000 and 2015 was 24.5% to 100% in goats, 8.87% to 100% in sheep, 11.4% to 24.4% in buffaloes, and 3% to 66.7% in cattle [21]. The prevalence of fascioliasis was also recorded in Pakistan, China, Saudi Arabia, Iraq, Iran, Russia, Turkey, Thailand, Nepal, Vietnam, Japan, Philippines, Korea, Cambodia, and Bangladesh in Asia. According to the results of 41 studies on 13 countries in Asia, the highest incidence during 2000–2015 was found in cattle (0.71–69.2%), and it was followed by buffaloes (2.08–68.0%), sheep (0.35–31.4%), and goats (0.0–47%). Three studies reported the incidence of fascioliasis during 2000–2015 in Papua New Guinea and Australia; it was the highest in cattle (26.5–81%) and the lowest in sheep (5.5–52.2%). Further, 23 studies reported data for 11 countries in Europe for 2000–2015; the highest prevalence was found in cattle (0.12–86%), and the lowest prevalence was found in goats (0.0–0.8%) [21]. For Africa, 31 studies reported data for 2000–2015; the incidence was the highest in cattle (1.2–91%) and the lowest in sheep (0.19–73.7%) [21]. In Iran, which is a neighboring country of Pakistan, the mean prevalence reported for the period 2000–2016 was 4.2% in cattle, 2.4% in goats, 2% in sheep, and 21% in buffaloes [22].

Fascioliasis has been reported as most prevalent in the densely populated province of Punjab, Pakistan [23]. The majority of the population is dependent on the rearing of livestock, including cattle, buffaloes, sheep, and goats [23]. Climatic variations serve as a key factor for the spread of fascioliasis, the incidence of which varies on both a monthly and annual basis [24,25]; in dry climatic conditions, a significantly lower number of fascioliasis infections are observed in Pakistan [24]. Although many studies in the literature address fascioliasis in humans and livestock species from Pakistan, the exact prevalence of this disease is still unknown. By considering the epidemiological aspects of the fascioliasis burden in humans and animals at national and international levels, the health and economic burden of this infection can be calculated. This will help the creation and implementation of preventive programs against the disease which will prove useful in the future from economic and health perspectives.

The present systematic review aimed to determine the distribution pattern and prevalence of fascioliasis among livestock and humans in various endemic areas of Pakistan in the period from 2000 to 2020.

## 2. Materials and Methods

### 2.1. Data Collection and Extraction

**Figure 1:**
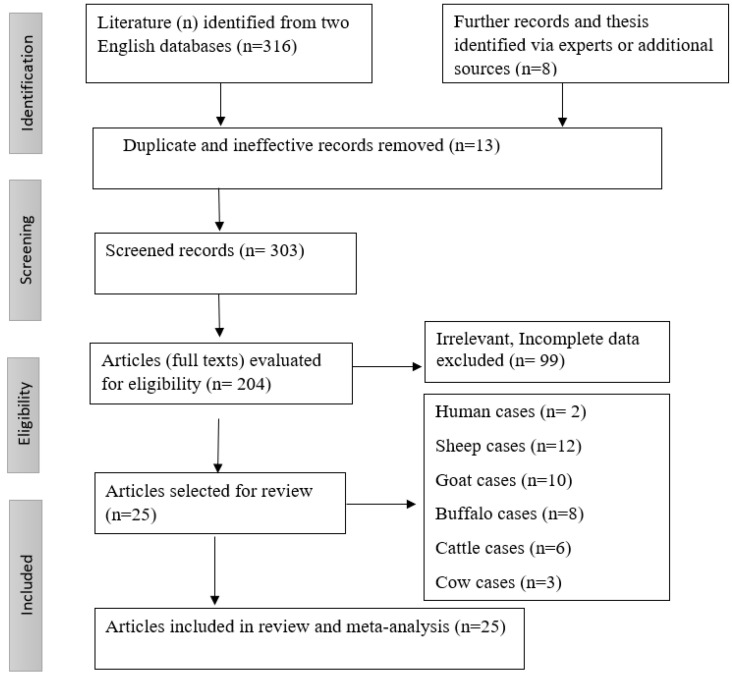
PRISMA flowchart describing the study design process.

We searched the published literature on the prevalence of fascioliasis in livestock and in human hosts in Pakistan and identified the 30 most relevant articles published between 2000 and 2020 (Table 1). More studies were published in 2012 than in the other years between 2000 and 2020, as shown in Table 1 and Figure 1. All the data were collected and extracted by the three authors in order to determine the prevalence rate of fascioliasis in Pakistan.

### 2.2. Search Strategy

The keywords “prevalence”, “fascioliasis”, “Pakistan”, “humans, “livestock”, “cattle”, “buffaloes”, “goats”, “cow”, and “sheep” were used either separately or in combination to search for relevant articles published in the period of 2000–2020.

### 2.3. Inclusion and Exclusion Criteria

For this meta-analysis, original articles and epidemiological studies on fascioliasis in humans and livestock were selected and examined thoroughly to identify those that reported the infection rate in Pakistan. Incomplete studies (for example, articles with abstracts only), irrelevant studies (for example, articles that did not report any Fasciola species diseases), duplicate studies, and articles that could not be accessed were excluded.

### 2.4. Databases

The English-language databases PubMed, Google Scholar, ScienceDirect, and Scopus were searched.

### 2.5. Publication Bias and Heterogeneity Assessment

**Figure 2:**
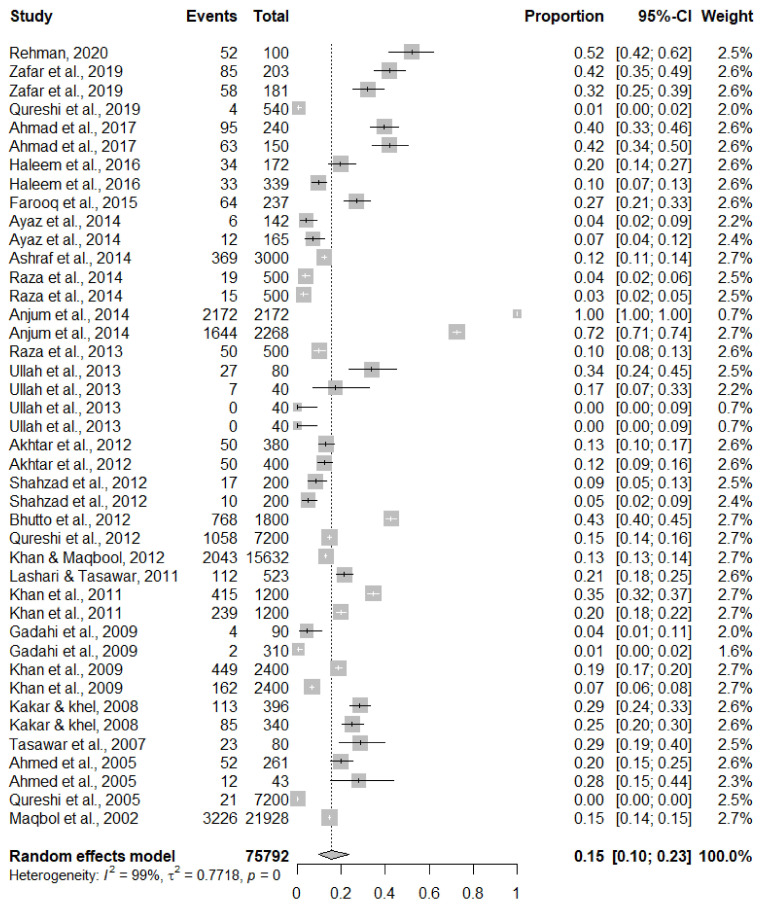
Forest plot diagram depicting the prevalence of fascioliasis in human and domestic animals [24,25,26,27,28,29,30,31,32,33,34,35,36,37,38,39,40,41,42,43,44,45,46,47,48,49].

**Figure 3:**
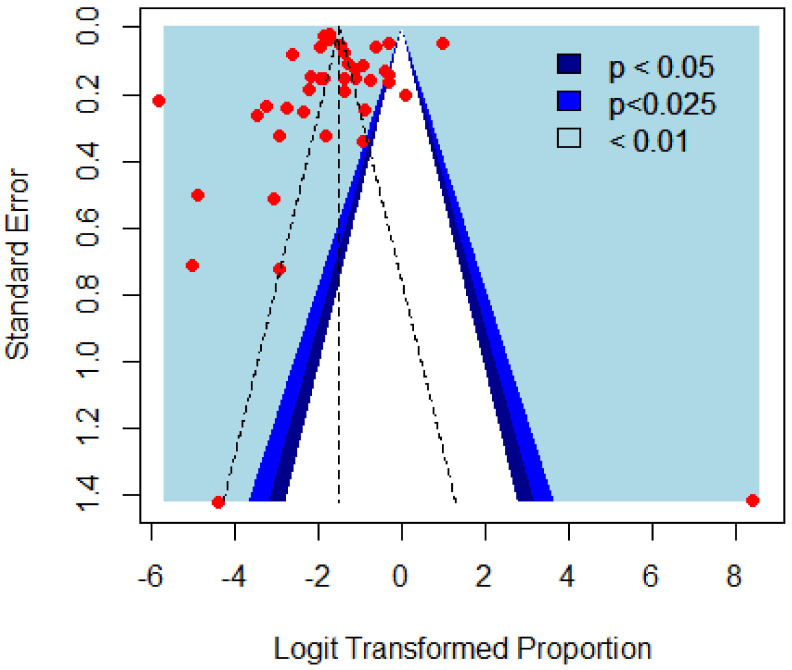
Funnel plot for studies included in the meta-analysis.

The confidence interval and effect size for each study was calculated and is represented by the forest plot in Figure 2. Potential publication biases in the selected studies were analyzed with the help of a funnel plot (Figure 3) and Egger’s test. The I^2^ index, tau^2^, and Cochran’s Q test were used for incoherence and heterogeneity assessment. If heterogeneity was detected, we used the random-effects model and fixed-effects model to estimate heterogeneity among the selected studies. For the random-effects model, the DerSimonian–Laird (1986) estimate was used, and, for adjustment of the test results and confidence interval (CI), the Hartung and Knapp (2003) correction was used.

## 3. Results

### 3.1. Fascioliasis in Humans

Two studies—one each from Punjab and Khyber Pakhtunkhwa (KPK)—reported fascioliasis in humans (Table 1). Based on the data of these studies, the overall prevalence in humans was 0.3 (Table 2). According to the provisional distribution data, the prevalence was higher in KPK (0.7%) than in Punjab (0.3%) between 2000 and 2020, as shown in Table 3. In KPK, *F. hepatica* was analyzed, while, in Punjab, both *F. hepatica* and *F. gigantica* were analyzed.

### 3.2. Fascioliasis in Sheep

A total of 12 studies from four provinces, namely, Punjab, KPK, Baluchistan, and ICT, reported data on fascioliasis in sheep (Figure 1). The overall prevalence in sheep was 53.5%, as shown in Table 2. According to the provisional distribution data, Punjab had the highest prevalence 65.7%, and it was followed in descending order by Baluchistan 19.9%, KPK 15.7% and ICT 4.4% (Table 3).

### 3.3. Fascioliasis in Goat

Ten studies reported fascioliasis data on goats in 2000–2020 from four provinces—Punjab, KPK, Baluchistan, and ICT (Figure 1). The overall prevalence was 44.9%, as shown in Table 2. According to the provisional distribution data, Punjab had the highest prevalence 53.3%, and it was followed by Baluchistan 27.9%, KPK 11.4%, and ICT 0.6% (Table 3).

### 3.4. Fascioliasis in Buffalo

Nine studies on the incidence of fascioliasis in buffaloes were conducted in four provinces, namely, Punjab, KPK, Sindh, and Baluchistan, during the period 2000–2020, as shown in Figure 1. The overall prevalence in buffaloes was 16.8% (Table 2). According to the provisional distribution data, Sindh had the highest prevalence 42.7%, and it was followed by Baluchistan 25%, KPK 15.9% and Punjab 15.4%, as shown in Table 3.

### 3.5. Fascioliasis in Cattle

Five studies on cattle data were conducted in Punjab and one study in KPK in the period 2000–2020 (Figure 1). The overall prevalence was 12.7% (Table 2), as well as the prevalence in Punjab was 12.8% and KPK 5.9%, as shown in Table 3.

### 3.6. Fascioliasis in Cow

A total of four studies from KPK and Baluchistan conducted between 2000 and 2020 reported the fascioliasis incidence in cows (Figure 1). The overall prevalence was 21.3%. Baluchistan had a higher prevalence 28.5% than KPK 13.3%, as shown in Table 3.

### 3.7. Publication Bias and Heterogeneity

According to the results of Egger’s test (0.161), with the exception of the studies on goats, there was no publication bias in any of the other studies (Figure 3). Therefore, overall, there was no publication bias in the literature included in this review (Figure 3). Further, Egger’s test was used for the analysis of publication bias in each subgroup, as shown in Table 4. Subgroup analysis was conducted because there was high heterogeneity between all the studies (I^2^ = 99.7%) (Table 3), but the heterogeneity was not significant for studies conducted on fascioliasis in human hosts (I^2^ = 65.8%) and studies from Baluchistan (I^2^ = 74.3%).

## 4. Discussion

The present study reviews data on the prevalence of fascioliasis in humans and domestic livestock in Pakistan. Across all the reviewed studies, 13,720 samples from all hosts were positive for *Fasciola* spp. With regard to the prevalence of fascioliasis in different animal hosts (sheep, goats, buffaloes, cattle, and cows), sheep exhibited the highest prevalence overall in Pakistan based on data collected between 2000 and 2020 (Table 1). According to the provisional distribution data, the province of Punjab had a higher number of infected sheep than other domestic livestock [50]. Snails serve as the intermediate hosts for the transmission of Fasciola sp. Floods, rain, and irrigation systems support snails [51], and this may lead to the outbreak of fasciolosis in sheep and cattle under such environmental conditions. These favorable conditions predominantly exist throughout the Punjab provinces and are conducive for the transmission of *Fasciola* sp. [23,24], except in the northern highlands of the region, where decreased infections were encountered [23].

According to the provisional distribution data, Sindh had an overall higher incidence of fascioliasis in buffaloes than in other livestock animals between 2000 and 2020. A study [52] from another Asian country, Bangladesh, reported that Satkania in the Chittagong district is the most vulnerable place for infections (50%) caused by trematode parasites. Similar to the present findings, other studies reported the incidence of Fasciola spp. in buffaloes as 44.70% [53] and 50.0% [54] in Hunan Province, China, and Southern Mindanao, Phillipines, respectively. *F. gigantica* is the main infectious agent in tropical, irrigated lowland rice fields [55], which are characteristic of the Sindh province. This may explain the higher infection rate in buffaloes in this province. One of the studies conducted in Sindh found that the prevalence of fascioliasis was higher in winter than in summer because the rate of infection increases with the rate of humidity in environment [39], and similar observations were reported in another study [56].

Fascioliasis in livestock results in remarkable economic losses worldwide. Globally, liver infection with Fasciola spp. is estimated to result in losses of USD 3.2 billion per annum in the livestock industry [21]. Research on the prevention of fascioliasis transmission could help counter such economic losses in the industry. Movement of animals is frequent in Pakistan, and this might play a role in high parasite gene flow [57]. Accordingly, one study reported that transportation of livestock animals contributes to high gene flow, new parasites, and exposure of hosts to various parasite populations [26]. The current review provides an overview of the distribution of fascioliasis according to host species and geographical location and may, therefore, be useful for understanding the patterns of transmission. Based on the epidemiological data, measures could be developed and implemented to prevent the transmission of fascioliasis.

Some of the limitations of the present review are the low number of studies on humans and a few other host animals, such as cows and cattle, the lack of standard diagnostic techniques used to measure prevalence, the lack of data on risk factors, and insufficient data regarding intermediate hosts such as snails (which were not included in the present study). These limitations could have resulted in a bias in the calculation of the prevalence of fascioliasis in host species in Pakistan.

## 5. Conclusions

Fascioliasis is a neglected, global zoonotic disease that is prevalent among buffaloes and sheep in Pakistan. The data indicate the threat posed to human health and economic losses in underdeveloped areas. Based on the data, precautionary measures, such as increased screening for fascioliasis, provision of clean drinking water, safe water irrigation in agricultural fields, and preventive treatment of animals in endemic areas, might help decrease the incidence of fascioliasis in Pakistan.

## Figures and Tables

**Table 1:** Summary of the main characteristics of the studies included in the systematic review and meta-analysis.

Province	Species	Host	Detection Method	No. of Samples Examined	No. of Positive Samples	Proportion of Positive Samples	Reference
Khyber Pakhtunkhwa (KPK)	*Fasciola* spp.	Sheep	FM, Mic	100	52	0.52	[26]
Punjab	*F. hepatica*	Sheep	ELISA	203	85	0.4187	[27]
Punjab	*F. hepatica*	Goat	ELISA	181	58	0.3204	[27]
KPK	*F. hepatica*	Human	DS, Mic	540	4	0.0074	[28]
Punjab	*Fasciola* spp.	Goat	Sed, MT, Mic	240	95	0.3958	[29]
Punjab	*Fasciola* spp.	Sheep	Sed, MT, Mic	150	63	0.42	[29]
KPK	*F. hepatica*	Cow	DS, FF	172	24	0.1395	[30]
KPK	*F. gigantica*	Cow	DS, FF	172	10	0.0581	[30]
KPK	*F. hepatica*	Sheep	DS, FF	339	20	0.059	[30]
KPK	*F. gigantica*	Sheep	DS, FF	339	13	0.0383	[30]
Punjab	*F. hepatica*	Cattle	Sed, Mic	237	39	0.1646	[31]
Punjab	*F. gigantica*	Cattle	Sed, Mic	237	20	0.0844	[31]
KPK	*F. hepatica*	Cattle	PCR, Mic	307	18	0.0586	[32]
Punjab	*F. hepatica*	Buffalo	PCR, Mic	3000	369	0.123	[33]
Punjab	*F. hepatica*	Goat	Sed, FM	500	16	0.032	[34]
Punjab	*F. gigantica*	Goat	Sed, FM	500	3	0.006	[34]
Punjab	*F. hepatica*	Sheep	Sed, FM	500	11	0.022	[34]
Punjab	*F. gigantica*	Sheep	Sed, FM	500	4	0.008	[34]
Punjab	*Fasciola* spp.	Sheep	FF	2172	1032	0.4751	[35]
Punjab	*Fasciola* spp.	Sheep	FF	2172	1140	0.5249	[35]
Punjab	*Fasciola* spp.	Goat	FF	2268	703	0.31	[35]
Punjab	*Fasciola* spp.	Goat	FF	2268	941	0.4149	[35]
Punjab	*F. hepatica*	Cattle	Sed, Mic, FM	500	45	0.09	[35]
Punjab	*F. gigantica*	Cattle	Sed, Mic, FM	500	5	0.01	[35]
KPK	*F. hepatica*	Buffalo	PM	80	11	0.1375	[36]
KPK	*F. hepatica*	Cow	PM	40	2	0.05	[36]
KPK	*F. hepatica*	Sheep	PM	40	0	0	[36]
KPK	*F. hepatica*	Goat	PM	40	0	0	[36]
KPK	*Fasciola* spp.	Sheep	FS, EC, Sed	380	50	0.1316	[37]
KPK	*Fasciola* spp.	Goat	FS, EC, Sed	400	50	0.125	[37]
Punjab	*F. hepatica*	Sheep	PCR, Bile samples	200	17	0.085	[38]
Punjab	*F. hepatica*	Goat	PCR, Bile samples	200	10	0.05	[38]
Sindh	*F. gigantica*	Buffalo	DS, Sed	1800	768	0.4267	[39]
Punjab	*Fasciola* spp.	Buffalo	Mic	7200	1058	0.1469	[24]
Punjab	*F. gigantica*	Cattle	PM, DS, Sed, FM	15,632	2043	0.1307	[40]
Punjab	*F. hepatica*	Sheep	Sed, FM, Mic	523	112	0.2141	[41]
Punjab	*Fasciola* spp.	Buffalo	MT, EC	1200	415	0.3458	[42]
Punjab	*Fasciola* spp.	Cattle	MT, EC	1200	239	0.1992	[42]
ICT	*Fasciola* spp.	Sheep	Sed, FM, Mic	90	4	0.0444	[43]
ICT	*Fasciola* spp.	Goat	Sed, FM, Mic	310	2	0.0065	[43]
Punjab	*F. hepatica*	Buffalo	EE	2400	16	0.0067	[44]
Punjab	*F. gigantica*	Buffalo	EE	2400	433	0.1804	[44]
Punjab	*F. hepatica*	Cattle	EE	2400	57	0.0238	[44]
Punjab	*F. gigantica*	Cattle	EE	2400	105	0.0437	[44]
Baluchistan	*F. hepatica*	Cow	PM	396	64	0.1616	[45]
Baluchistan	*F. gigantica*	Cow	PM	396	49	0.1237	[45]
Baluchistan	*F. hepatica*	Buffalo	PM	340	39	0.1147	[45]
Baluchistan	*F. gigantica*	Buffalo	PM	340	46	0.1353	[45]
Punjab	*F. hepatica*	Goat	FM, Sed	80	23	0.2875	[46]
Baluchistan	*F. hepatica*	Sheep	PM	261	20	0.0766	[47]
Baluchistan	*F. hepatica, F. gigantica*	Sheep	PM	261	32	0.1226	[47]
Baluchistan	*F. hepatica*	Goat	PM	43	3	0.0698	[47]
Baluchistan	*F. hepatica, F. gigantica*	Goat	PM	43	9	0.2093	[47]
Punjab	*F. hepatica, F. gigantica*	Human	FF	7200	21	0.0029	[48]
Punjab	*F. gigantica*	Buffalo	FM, Sed	21,928	3226	0.1471	[49]

FM = flotation method; Mic = microscope; DS = direct smear; Sed = sedimentation; MT; McMaster technique; FF = fecal flotation; PM = postmortem; FS = fresh smear; EC = egg count; EE = egg examination.

**Table 2:** Prevalence of fasciolosis according to host and geographical location in Pakistan.

Type	Host/Province	No. of Studies	No. of Samples Examined	No. of Positive Samples	Prevalence
Host	Human	2	7740	25	0.3%
	Sheep	12	4958	2655	53.5%
	Goat	10	4262	1913	44.9%
	Buffalo	9	38,113	6409	16.8%
	Cattle	6	20,276	2571	12.7%
	Cow	4	750	160	21.3%
	Overall	41	76,099	13,738	18.1%
Region	Punjab	15	70,114	12,409	17.7%
	KPK	6	2745	293	10.7%
	Baluchistan	2	1040	262	25.2%
	Sindh	1	1800	768	42.7%
	ICT	1	400	6	1.5%
	Overall	25	76,099	13,738	18.1%

**Table 3:** Geographical distribution and prevalence of *Fasciola* spp. in different hosts.

Province	Host	No. of Studies	No. of Samples Examined	No. of Positive Samples	Prevalence
Punjab	Human	1	7200	21	0.3%
	Sheep	6	3748	2464	65.7%
	Goat	6	3469	1849	53.3%
	Buffalo	5	35,728	5517	15.4%
	Cattle	5	19,969	2553	12.8%
	Total	-	70,114	12,409	17.7%
Khyber Pakhtunkhwa	Human	1	540	4	0.7%
	Sheep	4	859	135	15.7%
	Goat	2	440	50	11.4%
	Buffalo	2	245	39	15.9%
	Cow	3	354	47	13.3%
	Cattle	1	307	18	5.9%
	Total	-	2745	293	10.67%
Baluchistan	Sheep	1	261	52	19.9%
	Goat	1	43	12	27.9%
	Buffalo	1	340	85	25.0%
	Cow	1	396	113	28.5%
	Total	-	1040	262	25.2%
Sindh	Buffalo	1	1800	768	42.7%
ICT	Sheep	1	90	4	4.4%
	Goat	1	310	2	0.6%
Overall		43	76,099	13,738	18.1%

ICT; Islamabad capital territory.

**Table 4:** Subgroup meta-analysis for comparison of the prevalence of *Fasciola* spp. in humans and domestic livestock in various geographical regions of Pakistan.

Characteristics	Host/Province	No. of Studies	Heterogeneity Factors		Heterogeneity	Publication Bias
			I^2^ (%)	tau^2^	Q	Egger’s Test Result
Human	Human	2	65.8	0.288	2.93 ^n.s.^	-
Animals	Sheep	12	96.6	1.032	320.2 **	−0.300 ^n.s.^
	Goat	10	99.0	2.866	928.8 **	−4.29 **
	Buffalo	9	99.4	0.336	1170 **	1.38 ^n.s.^
	Cattle	5	97.7	0.221	171.1 **	0.148 ^n.s.^
	Cow	4	89.8	0.451	29.3 **	−2.45 ^n.s.^
	Overall	39	99.2	0.712	5036 **	0.752 ^n.s.^
Regions of Pakistan	Punjab	15	99.8	1.213	7475 **	0.303 ^n.s.^
	KPK	6	96.6	0.867	145.1 **	−0.491 ^n.s.^
	Baluchistan	2	74.3	0.038	3.89 ***	-
	Sindh	1	-	-	-	-
	ICT	1	-	-	-	-
	Overall	25	99.7	1.16	8316 **	0.161 ^n.s.^

n.s. = non-significant. ** *p* < 0.01; *** *p* < 0.001.

## Data Availability

Not applicable.
